# Supplementary material for: Rapid Machine Learning-Driven Detection of Pesticides and Dyes Using Raman Spectroscopy
Source: J Chem Inf Model. 2026 Mar 17;66(7):3803–13. doi: 10.1021/acs.jcim.6c00396 (PMC13080974; doi:10.1021/acs.jcim.6c00396)
Supplement: Supplementary file 1 [file ci6c00396_si_001.pdf]

Supporting Information: Rapid Machine Learning–Driven Detection of Pesticides and Dyes Using Raman Spectroscopy

Quach Thi Thai Binh,[†,‡] La Thuan Phuoc,[†,‡] Pham Xuan Hai,[†,‡] Thang Bach Phan,[¶,§,‡] Vu Thi Hanh Thu,[*,†,‡] and Nguyen Tuan Hung[*,‖,†,‡]

†*Faculty of Physics and Physics Engineering, University of Science, Ho Chi Minh City 700000, Viet Nam*

‡*Vietnam National University, Ho Chi Minh City 700000, Viet Nam*

¶*Advanced Materials Technology Institute Vietnam National University Ho Chi Minh City (formerly affiliated with Center for Innovative Materials and Architectures), Ho Chi Minh City, 700000, Viet Nam*

§*University of Health Sciences (UHS), Viet Nam National University Ho Chi Minh City, Ho Chi Minh City, 700000, Viet Nam*

‖*Department of Materials Science and Engineering, National Taiwan University, Taipei 10617, Taiwan*

E-mail: vththu@hcmus.edu.vn; nguyenth@ntu.edu.tw

Table of contents:

- **Table S1**: Comparison of the CPU and GPU efficiencies.

- **Figure S1**: Comparison of validation accuracy among different CNN backbones.

Table S1: Comparison of the CPU and GPU efficiencies for MLRaman with 25 epochs.

PC	Device	Total training time	Average time per epoch
1	NVIDIA GeForce RTX 4090	5.07 min	12.17 s
	AMD Ryzen Threadripper 3990X	15.21 min	36.49 s
2	NVIDIA GeForce RTX 3060	4.62 min	11.10 s
	AMD Ryzen 9 5900X	15.74 min	37.77 s
3	NVIDIA GeForce RTX 2060	14.57 min	29.13 s
	Intel Core i5-11400F	41.14 min	98.74 s

In Table S1, we show the comparison in terms of both the CPU and GPU, in which we used three workstations with different CPU/GPU for testing, including RTX 4090/AMD Ryzen Threadripper 3990X, RTX 3060/AMD Ryzen 9 5900X, and RTX 2060/Core i5-11400F. Compared with the GPU, the CPU takes almost three times as long.

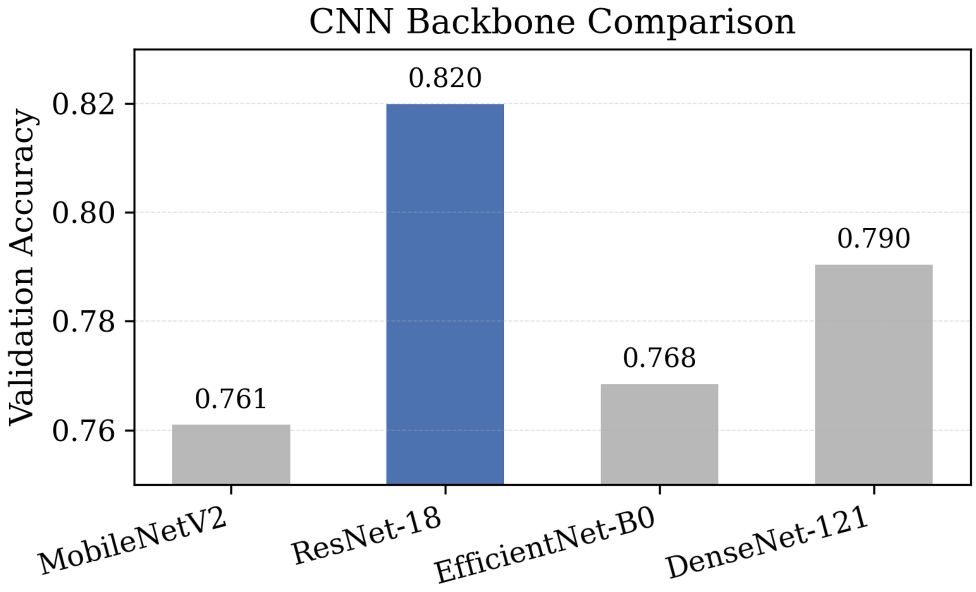

Figure S1: Comparison of validation accuracy obtained using different CNN backbones.

In Figure S1, we show the validation accuracy comparison among different CNN backbones evaluated in this study. Among the tested models, ResNet-18 achieves the highest

validation accuracy, outperforming MobileNetV2, EfficientNet-B0, and DenseNet-121. This result indicates that ResNet-18 provides a more effective feature representation for the classification task.
